# Dynamic optimization of shared storage for renewable driven grids with insights from Egypt

**DOI:** 10.1038/s41598-025-32005-x

**Published:** 2025-12-26

**Authors:** Marwa Hassan, Eman Beshr

**Affiliations:** Arab Academy for Science, Technology & Maritime Transport, Cairo, 11865 Egypt

**Keywords:** Environmental sciences, Risk factors, Energy science and technology

## Abstract

This paper presents an adaptive Shared Energy Storage (SES) framework tailored to Egypt’s renewable energy landscape. The proposed approach integrates dynamic SES partitioning, Nash bargaining–based cooperation, and a distributed ADMM optimization algorithm to support flexible, real-time leasing of centralized SES units by multiple renewable producers. Unlike conventional fixed-allocation or fully cooperative models, the framework adjusts storage access in response to grid demand, market prices, and forecast variations. Simulation results indicate that the adaptive SES framework can increase storage utilization by more than 40% compared with fixed allocation, while maintaining economic viability under forecast uncertainty and battery degradation. Under $\pm 10\%$ prediction errors or reduced round-trip efficiency, the system continues to provide stable operation and balanced economic outcomes. The Nash bargaining mechanism facilitates equitable benefit sharing between producers and the SES operator, and the distributed ADMM algorithm enables scalable, near–real-time coordination. By coupling cooperative game theory with adaptive control, this study introduces a market-aware SES model that can enhance renewable integration, support grid reliability, and improve economic resilience in emerging energy systems. The framework aligns with Egypt’s Vision 2035 priorities and offers insights for policymakers and grid operators considering SES deployment at scale.

## Introduction

Energy storage has become a cornerstone technology in modern power systems, playing a crucial role in the seamless integration of renewable energy sources, enhancing grid stability, and accelerating the transition to sustainable energy solutions. In Egypt, the rapid expansion of renewable energy projects, such as the Benban Solar Park and the Gulf of Suez Wind Farms, highlights the urgent need for efficient energy storage solutions to manage variability and ensure a stable power supply. However, traditional energy storage systems (ESS) are often deployed in a fragmented manner, serving individual power generation companies, grid operators, or end-users, leading to suboptimal utilization and increased capital and maintenance costs. Egypt’s renewable energy sector has witnessed significant growth, driven by ambitious projects and strategic initiatives to enhance energy security and sustainability. The New and Renewable Energy Authority (NREA) has driven Egypt’s clean energy transformation through projects such as Benban Solar Park and the Zafarana and Jabal al-Zeit wind farms, demonstrating national progress toward large-scale renewable integration^1^. However, the absence of large-scale storage integration continues to limit dispatch flexibility. Complementary national reports from the Egyptian Electricity Holding Company (EEHC) and Ministry of Electricity and Renewable Energy (MOEE) confirm that grid modernization and smart control infrastructure remain under development^2,3^. Egypt’s participation in the EuroAfrica Interconnector positions it as a regional energy hub connecting Africa, Europe, and the Middle East^4^, yet cross-border storage and balancing protocols remain underexplored. Broader studies such as the World Bank’s Pan-Arab Electricity Market framework^5^, IRENA’s *Renewable Energy Outlook: Egypt*^6^, and LAS’s regional initiatives^7^ underscore strong policy alignment but lack quantitative mechanisms for shared storage coordination. Similarly, macro-regional initiatives like DESERTEC^8,9^ highlight transcontinental renewable trade potential but omit local-level operational optimization and market-sharing mechanisms.

To address variability and ensure reliability, Shared Energy Storage (SES) has emerged as a promising distributed mechanism for cooperative balancing. Zheng *et al.*^10^ modeled peer-to-peer trading embedded with shared residential storage but neglected regulatory and communication delays. Qian *et al.*^11^ developed a Nash game model for SES pricing, though assuming perfect information exchange among users. Xu *et al.*^12^ introduced asymmetric Nash bargaining for multi-user coordination but overlooked dynamic infrastructure constraints. Meng *et al.*^13^ extended Nash bargaining to multi-energy prosumers, yet their framework presumes full cooperation without transaction uncertainty. Wang *et al.*^14–16^ proposed multi-agent and joint optimization strategies for cooperative energy management, but most rely on ideal market access and centralized coordination. Qiao *et al.*^17^ applied Nash negotiation to multi-microgrid alliances, though computational scalability and communication latency were not addressed.

Recent research has emphasized robust and intelligent optimization to improve SES reliability under uncertainty. Dong *et al.*^18^ proposed a hybrid probabilistic–IGDT model for sustainable communities but did not incorporate real-time market feedback. Dorahaki *et al.*^19^ enhanced flexibility via robust optimization, though with high computational overhead for real deployment. Ghasemnejad *et al.*^20^ integrated hydrogen storage and thermal comfort in prosumer communities but focused narrowly on building-scale systems. Li *et al.*^21^ introduced multi-time-scale scheduling for virtual power plants with energy storage; however, implementation assumes deterministic load forecasts. Dolatabadi *et al.*^22^ developed distributed market coordination but did not quantify communication delays or latency effects. Qiu *et al.*^23^ used distributionally robust optimization for hydrogen–electric coordination yet omitted cross-regional energy sharing. Zhu *et al.*^24^ analyzed robust sizing under power quality constraints but neglected cooperative dispatch and dynamic participation.

Foundational community energy storage (CES) works have demonstrated economic feasibility but within simplified or static conditions. Sardi *et al.*^25^ assessed CES planning via cost–benefit analysis but excluded stochastic renewable variations. Parra *et al.*^26^ studied PV energy time-shifting but assumed fixed pricing and perfect forecasts. Zakeri and Syri^27^ performed lifecycle cost comparisons of storage technologies without cooperative frameworks. Díaz-González *et al.*^28^ reviewed wind power storage applications but did not address shared-use models. De Sisternes *et al.*^29^ and Go *et al.*^30^ quantified storage value in decarbonized grids but ignored multi-agent coordination and market fairness.

Another example , AI-driven methods are expanding SES adaptability but remain limited in interpretability and physical validation. Wilk *et al.*^31^ applied multi-agent reinforcement learning for smart community control but without considering real-time grid constraints. Li *et al.*^32^ reviewed shared storage pricing and auxiliary services but noted a lack of adaptive algorithms for uncertainty. Liu *et al.*^33^ proposed dynamic game-based energy sharing among PV prosumers, yet validation under heterogeneous infrastructure remains limited. Egypt recently awarded its first contract for a standalone utility-scale battery storage project, marking a new milestone in the nation’s grid modernization and energy storage deployment . To address these challenges, Shared Energy Storage (SES) has emerged as a promising alternative, allowing multiple entities to share energy storage capacity dynamically, thereby improving grid stability, resource allocation, and economic efficiency. Recent years have witnessed a surge of research focused on the optimization, market integration, and robust operation of shared and community energy storage systems. For instance, Khorasany et al.^34^ proposed a probabilistic/information gap decision theory-based bilevel strategy for managing multi-carrier energy communities. However, their model assumes full system knowledge and lacks integration with real-time market dynamics. Wang *et al.*^35^ proposed a Stackelberg equilibrium-based energy management strategy for regional electricity–hydrogen markets, demonstrating effective hierarchical coordination between operators and participants, yet the framework does not explicitly address cooperative shared storage interactions across multiple entities. Wang et al.^35^ introduced a Stackelberg game-theoretic framework for community energy storage coordination, but its hierarchical design reduces flexibility for decentralized control. Dong et al.^36^ developed a flexibility-oriented model using a hybrid probabilistic-IGDT method for local multi-carrier communities, although it relies on predefined uncertainty bounds that may not fully capture fast-changing renewable outputs. Zhao et al.^37^ addressed renewable uncertainty in peer-to-peer trading using stochastic optimization; however, the scalability of their model for large, diverse communities remains limited. Liao et al.^38^ employed multi-agent reinforcement learning to optimize SES operations in local markets, yet such models often demand extensive training data and face convergence issues in dynamic environments. Li et al.^39^ proposed a robust optimization model for distributed SES under uncertainty, but it assumes static infrastructure and limited adaptability to evolving grid communication constraints. Zhou et al.^40^ examined peer-to-peer trading and SES operation in integrated electricity and gas networks, though their model is less applicable to single-carrier systems like Egypt’s. Wang et al.^41^ designed a robust scheduling approach for SES under multi-timescale uncertainties; however, the lack of cooperative coordination among independent agents limits its effectiveness in shared ownership contexts. Lin et al.^42^ proposed evolutionary algorithms for optimal storage siting and sizing, but their approach is largely confined to planning, without addressing real-time operation or adaptive coordination. More recent work continues to expand the SES design space. Khorasany et al.^43^ extended bilevel optimization using IGDT and probabilistic methods, but the absence of market-based leasing mechanisms limits its applicability in dynamic contexts. Wang et al.^44^ advanced Stackelberg models for hierarchical energy management, although their framework still assumes ideal communication links, which are unrealistic in developing energy networks. Similarly, Dong et al.^36^ introduced a hybrid probabilistic–IGDT approach to improve flexibility under uncertainty, yet their formulation does not address coordination or leasing across multiple stakeholders. Research in energy-sharing systems has increasingly focused on enhancing flexibility and resilience through robust and intelligent optimization frameworks. For example, a robust optimization model was developed to promote flexibility, self-sufficiency, and environmental sustainability in local multi-carrier energy communities^19^, while an IGDT-based optimization framework was introduced for prosumer-oriented citizen energy communities integrating hydrogen parking lots, energy sharing, and thermal comfort considerations^20^. Together, these studies emphasize uncertainty handling, multi-energy cooperation, and cross-sector integration, whereas the present work contributes by addressing adaptive shared-storage coordination and cooperative leasing mechanisms under realistic communication and infrastructure constraints. Building upon these efforts, this study introduces an adaptive, market-aware SES framework explicitly designed for Egypt’s renewable-rich energy system. The model integrates dynamic SES partitioning, Nash Bargaining-based cooperation, and distributed ADMM optimization to improve storage utilization, operational flexibility, and economic returns. Unlike prior SES models that assume static allocation or perfect cooperation, this study introduces a fully adaptive, market-aware optimization architecture that explicitly accounts for Egypt’s infrastructure limitations. The framework combines three methodological advances: (1) a *dynamic SES partitioning mechanism* that reallocates shared storage capacity in real time according to fluctuating demand and grid conditions; (2) a *Nash Bargaining–based cooperative model* that ensures fair, incentive-compatible leasing among heterogeneous renewable producers; and (3) a *distributed ADMM algorithm* that decomposes global coordination into local subproblems, enabling operation under partial communication and decentralized control. This integration allows the framework to function efficiently even when real-time data sharing and automation are constrained—conditions characteristic of Egypt’s evolving grid digitalization. Consequently, the proposed model enhances storage utilization, reduces curtailment, and strengthens economic performance, while demonstrating that cooperative SES management can remain viable within emerging markets with limited infrastructure readiness. The framework’s validity is confirmed through scenario-based simulations that show a 41.2% increase in SES utilization, 39.5% reduction in curtailment, and 58.95% improvement in renewable entity revenue. Robustness tests under forecast errors, infrastructure constraints, and battery degradation further affirm its practical viability. By aligning with Egypt’s Vision 2035 energy goals, this work provides actionable insights for policymakers, grid operators, and investors seeking scalable and resilient storage coordination strategies in renewable-driven markets.

While technically and economically feasible, full-scale implementation requires continued modernization of Egypt’s digital infrastructure, including smart metering, high-speed communication, and regulatory alignment—points elaborated in the *Limitations* section.

The paper is structured as follows: Section 1 presents the problem definition and literature review; Section 2 details the proposed methodology; Section 3 discusses the simulation results; Section 4 outlines limitations; and Section 5 concludes with key findings.

**Fig. 1:**
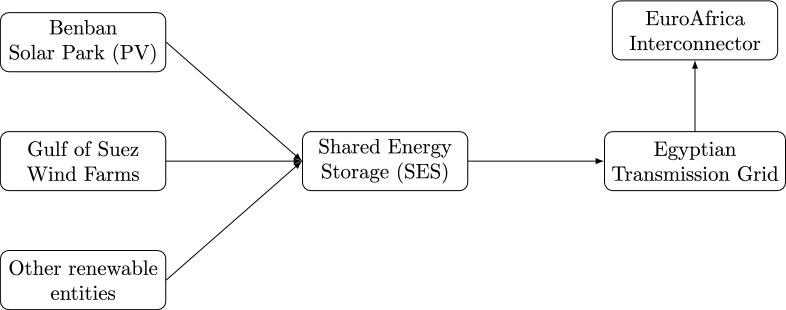
Conceptual layout of the case study, showing the interaction between Egypt’s major renewable entities, the shared energy storage (SES) station, the national transmission grid, and the EuroAfrica Interconnector.

Figure 1 presents the conceptual interaction between Benban Solar Park, the Gulf of Suez wind farms, the shared energy storage (SES) station, the transmission grid, and the EuroAfrica Interconnector.

## Methodology

This section outlines the proposed methodology for optimizing shared energy storage (SES) in Egypt’s renewable energy landscape, including adaptive allocation mechanisms, cooperative bargaining between SES operators and renewable producers, and an ADMM-based operational optimization model.

### Smart energy storage allocation strategy for SES

With the increasing reliance on renewable energy sources in Egypt, such as Benban Solar Park and the Gulf of Suez Wind Farms, integrating energy storage has become a key challenge. When multiple renewable energy plants share centralized Shared Energy Storage (SES) stations, efficient allocation of storage capacity is crucial to balance fluctuating supply and demand. Fixed storage allocation strategies lack flexibility, resulting in suboptimal utilization and higher operational costs. To address these limitations, this study proposes an adaptive partitioning strategy, which dynamically adjusts SES allocations based on real-time energy demand and market conditions. Unlike static models, this strategy enables renewable energy producers to lease SES capacity on an hourly basis, ensuring economic efficiency and operational flexibility. This adaptability allows SES stations to participate in electricity markets, optimize energy storage utilization, and reduce overall investment costs for renewable energy entities.This paper proposes a novel framework for shared energy storage (SES) management in renewable energy systems, combining adaptive allocation strategies, cooperative Nash bargaining for fair leasing, and a distributed ADMM optimization model to ensure efficient, market-aware operation.

#### Modular energy storage unit structure

**Fig. 2:**
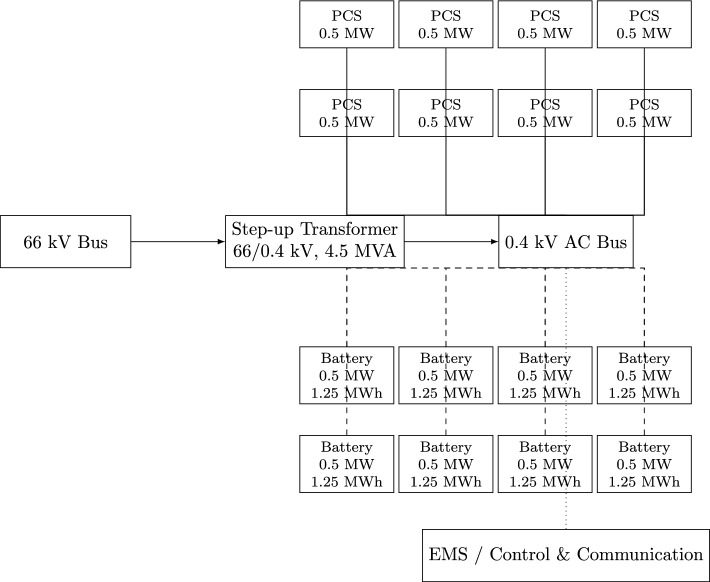
Compact modular SES unit (5 MW / 10 MWh) with 8 PCSs and 8 Battery Cabinets arranged in two rows.

To enhance flexibility, scalability, and responsiveness to the dynamic partitioning strategy, Shared Energy Storage (SES) stations are structured into modular units comprising energy storage battery cabinets and power conversion systems (PCSs). Each unit is rated at 5 MW power and 10 MWh storage capacity, making it compatible with Egypt’s 66 kV transmission network, which is the standard for high-capacity storage integration^34^. These values were selected based on a balance of grid stability requirements and cost optimization. The 5 MW power rating aligns with typical grid injection capacities for renewable energy plants in Egypt, ensuring minimal impact on grid frequency and voltage. The 10 MWh storage capacity provides a sufficient buffer to smooth out short-term fluctuations in renewable energy supply, allowing for effective participation in ancillary service markets. Each unit consists of a step-up transformer, eight battery cabinets, and eight PCSs, enabling independent operation and dynamic response to grid demands. By partitioning SES stations into standardized units, energy storage is optimized, scalable, and economically viable. This allows renewable energy producers, industrial consumers, and grid operators to efficiently lease storage capacity, minimizing idle storage and maximizing economic returns. This optimized usage improves Egypt’s energy market competitiveness and contributes to long-term sustainability, aligning with Egypt’s evolving energy policies and international best practices in shared energy storage. Figure 2 illustrates the compact modular SES unit, highlighting its internal configuration of PCSs and battery cabinets connected through a step-up transformer and supervised by the EMS. The dynamic allocation framework can readily incorporate additional features and evolving policies in Egypt.

#### Adaptive partitioning strategy for shared energy storage

Large-scale energy storage stations play a crucial role in stabilizing grids and integrating renewable energy sources, particularly in Egypt, where projects like Benban Solar Park and Gulf of Suez Wind Farms highlight the growing need for effective storage solutions. Conventional fixed storage allocation strategies often lead to inefficient utilization and increased operational costs. To address these challenges, this study proposes an adaptive partitioning strategy that allows centralized shared energy storage (SES) stations to dynamically allocate storage capacity in response to real-time energy demand and market conditions. Unlike static models, this approach enhances operational flexibility by dividing SES stations into independently managed zones, where storage capacity is dynamically adjusted based on hourly variations in energy supply, demand, and trading opportunities. These zones are virtually separated using a sophisticated control and communication system that monitors real-time data from renewable energy producers, grid operators, and market participants. This is achieved through a centralized energy management system (EMS) that collects data from smart meters and forecasting tools installed at each renewable energy plant and grid substation. The EMS uses this data to predict energy demand and generation, and then optimizes the allocation of storage capacity to each zone in real-time.This model assumes access to accurate and timely forecasts for renewable generation and demand. While this supports optimal SES allocation in our simulations, real-world forecast errors and uncertainty are likely to impact system performance. The communication infrastructure consists of a high-speed fiber optic network that connects all SES units, renewable energy plants, and grid substations, ensuring seamless data exchange and control.It should be noted that this framework assumes the availability of real-time market participation and continuous access to detailed grid and generation data. While such capabilities are essential for the optimal performance of adaptive SES allocation, they may not fully reflect the current realities of Egypt’s energy market, where market liberalization is ongoing, smart meter deployment is expanding, but comprehensive real-time data sharing and full market transparency have not yet been achieved. As such, our results should be interpreted as representing the upper-bound potential of SES under ideal conditions. The development of policies and future investments aimed at expanding smart grid infrastructure, market transparency, and digitalization will be crucial for enabling the full implementation of these strategies in practice. Through this strategy, renewable energy producers can lease SES capacity on a flexible basis, ensuring optimized energy dispatch while minimizing energy curtailment. The SES allocation process begins by collecting forecasted demand data from multiple renewable energy producers and determining the optimal storage distribution. For an SES station serving M renewable energy entities, including wind and solar power plants, the allocated storage for each entity is determined using the equation:

1 $$\begin{aligned} E_m^{\text {alloc}}(t) = \frac{E_m^{\text {demand}}(t)}{\sum _{i=1}^{M} E_i^{\text {demand}}(t)} \cdot E_{\text {total}} \end{aligned}$$

where $E_m^{\text {alloc}}(t)$ represents the allocated SES capacity for entity *m*, $E_m^{\text {demand}}(t)$ is the projected demand of entity *m*, and $E_{\text {total}}$ is the total available SES capacity at the centralized SES station. To ensure fair distribution and prevent overallocation, the total assigned capacity across all entities must not exceed the station’s maximum capacity, expressed as:

2 $$\begin{aligned} \sum _{m=1}^{M} E_m^{\text {alloc}}(t) \le E_{\text {SES}}^{\text {max}} \end{aligned}$$

Once the SES capacity is allocated, each entity can charge or discharge energy based on real-time grid conditions. The power available for charging and discharging at any given moment follows the constraints:

3 $$\begin{aligned} P_m^{\text {charge}}(t) = \alpha _m(t) \cdot P_m^{\text {max}}, \quad P_m^{\text {discharge}}(t) = (1 - \alpha _m(t)) \cdot P_m^{\text {max}} \end{aligned}$$

Here, $P_m^{\text {charge}}(t)$ and $P_m^{\text {discharge}}(t)$ denote the charging and discharging power of storage unit *m* at time *t*, respectively. $\alpha _m(t)$ is a binary decision variable equal to 1 during charging and 0 during discharging, while $P_m^{\text {max}}$ is the maximum power capacity. This constraint ensures that a storage unit cannot charge and discharge simultaneously.

4 $$\begin{aligned} \text {SoC}_k(t+1) = \text {SoC}_k(t) + \eta _c P_k^{\text {charge}}(t) - \frac{P_k^{\text {discharge}}(t)}{\eta _d} \end{aligned}$$

In this equation, $\text {SoC}_k(t)$ represents the state of charge of storage unit *k* at time *t*. $\eta _c$ and $\eta _d$ denote the charging and discharging efficiencies, respectively. The equation expresses the energy balance over time, showing how stored energy increases during charging and decreases during discharging, accounting for efficiency losses.

5 $$\begin{aligned} \text {SoC}_{\text {min}} \le \text {SoC}_k(t) \le \text {SoC}_{\text {max}} \end{aligned}$$

Here, $\text {SoC}_{\text {min}}$ and $\text {SoC}_{\text {max}}$ define the minimum and maximum permissible limits of the battery’s charge level. This constraint safeguards against deep discharge and overcharging, thus ensuring operational safety and battery longevity.

6 $$\begin{aligned} \sum _{m=1}^{M} P_m^{\text {market}}(t) + P_{\text {spot}}(t) \le P_{\text {SES}}^{\text {max}} \end{aligned}$$

In this constraint, $P_m^{\text {market}}(t)$ is the energy dispatched to the market by participant *m*, $P_{\text {spot}}(t)$ is the additional power sold in the spot market, and $P_{\text {SES}}^{\text {max}}$ is the total marketable capacity of the SES station. It ensures that the total traded energy from all participants does not exceed the station’s physical and regulatory capacity.

By implementing this adaptive SES partitioning framework, Egypt’s renewable energy market can achieve greater flexibility, increased economic efficiency, and enhanced grid stability. Compared to fixed storage allocation strategies, this dynamic model ensures that energy storage resources are utilized to their fullest potential, minimizing idle capacity while optimizing revenue generation. The strategy also facilitates cross-border electricity trading, supporting Egypt’s participation in regional energy markets such as the EuroAfrica Interconnector and the Egypt-Sudan power exchange initiative. Ultimately, this approach aligns with Egypt’s Vision 2035 for renewable energy expansion, ensuring a more resilient, cost-effective, and sustainable power system.In addition to the baseline coordination scenarios (fixed and adaptive SES), a third limited-infrastructure case (Scenario 3) was modeled to simulate SES operation under constrained control and communication, reflecting Egypt’s current grid maturity level.

### Cooperative energy storage allocation via nash bargaining

**Fig. 3:**
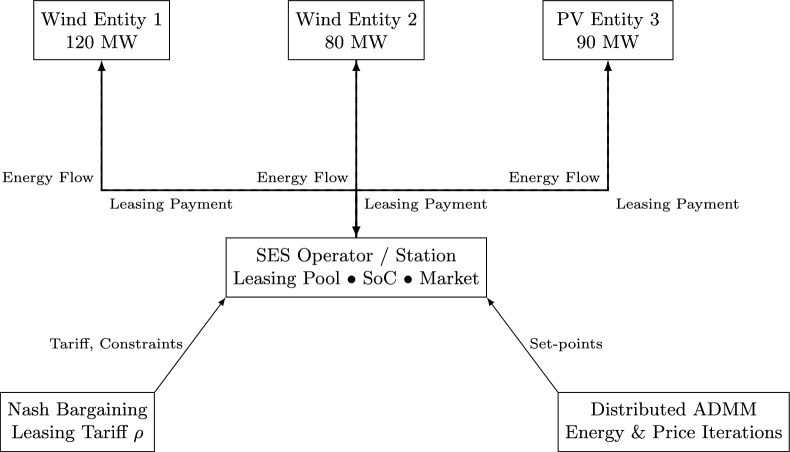
Nash Bargaining framework between SES Operator and multiple producers.

Following the adaptive allocation of Shared Energy Storage (SES), an optimized coordination framework is required to ensure fair and efficient utilization among multiple renewable energy stakeholders. A Nash bargaining approach is applied to determine storage allocation and pricing, balancing economic benefits for all participants. This framework consists of three main components: (1) renewable energy generator operations, (2) SES station management, and (3) a negotiation mechanism for leasing agreements. SES allocation is scheduled one day in advance, dynamically assigning capacity based on anticipated demand. Wind and solar power plants lease storage to stabilize power output, ensuring alignment with dispatch schedules and minimizing penalties. This approach enhances flexibility and prevents unnecessary curtailment of renewable energy. SES operators negotiate leasing terms, ensuring cost-effective pricing that supports renewable energy producers while maintaining SES profitability. Through Nash bargaining, equitable agreements are established, optimizing both utilization and economic returns. Any surplus SES capacity is integrated into electricity markets, participating in grid balancing services and frequency regulation. The self-scheduling mechanism ensures that available storage is used effectively, preventing underutilization and revenue losses. By leveraging a cooperative negotiation model, SES stations and renewable energy producers achieve optimal resource allocation, improved energy stability, and enhanced market participation, contributing to a more efficient and resilient energy system. Figure 3 depicts the overall system architecture, where renewable generation interacts with the SES and the grid, coordinated by the EMS using Nash bargaining and ADMM optimization

#### Renewable energy entity participation

The shared energy storage system (SES) enhances the ability of renewable energy producers to align their output with the grid, minimizing forecast deviations and curtailment while improving operational flexibility. The net benefit of a renewable energy producer $G_j$ is given by:

7 $$\begin{aligned} G_j = S_j - T_{j,s} - V_j^{\text {op}} - V_j^{\text {dev}} \end{aligned}$$

where $S_j$ represents revenue from energy sales, $T_{j,s}$ is the payment for storage leasing, $V_j^{\text {op}}$ is the operational and maintenance (O&M) cost, and $V_j^{\text {dev}}$ is the deviation penalty cost due to forecast inaccuracies. *This equation defines the producer’s net profit by subtracting leasing, operation, and deviation costs from total revenue.*

The revenue from energy sales is computed as:

8 $$\begin{aligned} S_j = \sum _{t=1}^{T} \eta _{\text {sell}}(t) P_j^{\text {del}}(t) \end{aligned}$$

where $\eta _{\text {sell}}(t)$ denotes the electricity price at time $t$, and $P_j^{\text {del}}(t)$ is the actual delivered power to the grid. *This captures the total energy sales revenue, reflecting variations in market price and power delivery.*

Deviation penalties apply when actual generation differs significantly from the scheduled generation:

9 $$\begin{aligned} V_j^{\text {dev}} = \zeta \sum _{t=1}^{T} \max \left( \left| P_j^{\text {act}}(t) - P_j^{\text {sch}}(t) \right| - \sigma P_j^{\text {sch}}(t), 0 \right) \end{aligned}$$

where $P_j^{\text {act}}(t)$ is the actual power generation, $P_j^{\text {sch}}(t)$ is the scheduled generation, $\sigma$ is the permissible deviation percentage, and $\zeta$ represents the penalty per unit deviation. *This penalizes producers when deviations exceed the allowable forecast margin, encouraging accurate scheduling.*

Renewable energy producers lease SES capacity to mitigate their variability. The leasing payment is determined as:

10 $$\begin{aligned} T_{j,s} = \rho _j \sum _{t=1}^{T} P_{j,s}(t) \end{aligned}$$

where $\rho _j$ is the leasing tariff per unit power, and $P_{j,s}(t)$ denotes the power exchanged between the producer and the SES at time $t$. *This quantifies the total payment producers make for using shared energy storage capacity.*

To ensure energy neutrality, the total exchanged power satisfies:

11 $$\begin{aligned} P_{j,s}(t) = -P_{s,j}(t) \end{aligned}$$

indicating that the energy injected into the SES by the renewable entity is equal to the energy retrieved. Consequently, the leasing payments between SES and renewable producers must satisfy:

12 $$\begin{aligned} T_{j,s} = -T_{s,j} \end{aligned}$$

ensuring balanced financial transactions. *These constraints maintain both energy and financial balance between producers and the SES, ensuring fair exchanges.*

#### Shared energy storage operation

The SES aims to maximize revenue through leasing agreements with renewable energy producers while also engaging in energy markets. The net benefit for the SES operator, denoted as $G_s$, is given by:

13 $$\begin{aligned} G_s = S_s^{\text {mkt}} + S_s^{\text {cap}} - \sum _{j} T_{j,s} - V_s^{\text {op}} \end{aligned}$$

where $S_s^{\text {mkt}}$ represents market trading revenue, $S_s^{\text {cap}}$ is the capacity service compensation, $\sum _{j} T_{j,s}$ accounts for leasing revenue from renewable producers, and $V_s^{\text {op}}$ denotes operational costs. *This defines the SES operator’s total profit, combining leasing and market income while subtracting operational costs.*

SES earns revenue from market participation by trading excess capacity:

14 $$\begin{aligned} S_s^{\text {mkt}} = \sum _{t=1}^{T} \gamma _{\text {mkt}}(t) P_s^{\text {mkt}}(t) \end{aligned}$$

where $\gamma _{\text {mkt}}(t)$ is the spot market price at time $t$, and $P_s^{\text {mkt}}(t)$ represents the SES energy dispatched to the market. *This reflects income generated from SES energy trading activities.*

Additionally, SES may receive compensation for maintaining energy storage availability:

15 $$\begin{aligned} S_s^{\text {cap}} = \omega E_s^{\text {max}} \end{aligned}$$

where $\omega$ is the capacity compensation rate, and $E_s^{\text {max}}$ denotes the maximum SES storage eligible for compensation. *This represents additional revenue earned by providing reserve or standby storage services.*

SES incurs operational expenses proportional to energy usage:

16 $$\begin{aligned} V_s^{\text {op}} = \mu _s \sum _{t=1}^{T} P_s^{\text {op}}(t) \end{aligned}$$

where $\mu _s$ is the SES unit O&M cost, and $P_s^{\text {op}}(t)$ represents energy consumed for system operations. *This captures the variable O&M costs linked to SES operation and maintenance.*

#### Cooperative optimization framework

To ensure efficient and fair allocation of SES capacity, a cooperative bargaining model is introduced, allowing renewable energy producers and SES operators to negotiate optimal leasing terms. The system-wide optimization objective is:

17 $$\begin{aligned} \max \left( F_j + F_s \right) \end{aligned}$$

where:

18 $$\begin{aligned} F_j = G_j + T_{j,s} \end{aligned}$$

19 $$\begin{aligned} F_s = G_s + \sum _{j} T_{s,j} \end{aligned}$$

ensuring collective economic efficiency while accounting for both renewable energy producers and SES entities. *This cooperative formulation aligns both parties’ interests to achieve a mutually beneficial outcome.*

The cooperation must meet minimum profit conditions to ensure fair participation:

20 $$\begin{aligned} G_j- G_j^0\ge 0, \quad G_s-G_s^0 \ge 0 \end{aligned}$$

where $G_j^0$ and $G_s^0$ are the baseline profit thresholds required for participation. *These constraints guarantee that each participant gains at least as much as in the non-cooperative case, ensuring incentive compatibility.*

By integrating dynamic pricing and real-time allocation, this framework enables a responsive SES system that optimally manages capacity leasing while adjusting to fluctuating market prices and renewable generation patterns.

This optimization model ensures that both SES operators and renewable energy producers achieve sustainable and profitable energy storage utilization, aligning with Egypt’s evolving energy market regulations.

#### Limitations of Nash bargaining approach

It should be acknowledged that the Nash bargaining framework applied in this study relies on several idealized assumptions. Most notably, it presumes that all participants behave rationally, possess full information, and report their preferences and data truthfully. In real-world energy markets, however, agents may act strategically, misreport information, or be influenced by bounded rationality and incomplete data access. Such behaviors could lead to suboptimal or unfair outcomes that diverge from the theoretical equilibrium. To address these challenges, future research should explore robust or stochastic game-theoretic models that explicitly account for information asymmetry, strategic misreporting, and the risk of irrational or adversarial behaviors among market participants. Incorporating mechanisms for incentive compatibility, distributed learning, or adaptive negotiation could further enhance the practical resilience of SES allocation strategies under real market conditions.Despite these limitations, the results presented here establish a crucial benchmark for SES performance in Egypt’s renewable energy transition. By highlighting both the opportunities and the practical challenges, this study provides a valuable foundation for further research and policy development toward a more resilient and efficient energy system.

### Optimization-based operational solution

To enhance the efficiency of shared energy storage (SES) utilization and ensure equitable profit distribution, a distributed Alternating Direction Method of Multipliers (ADMM) algorithm is employed. The ADMM framework effectively handles optimization problems with separable variables, ensuring strong convergence properties and robustness in energy trading. The optimization process is structured into two key subproblems: maximizing the profits of participating entities and negotiating the leasing payments for energy trading. To formulate the optimization problem, an auxiliary variable $\hat{X}_{i,j}(t)$ is introduced to represent the expected energy to be leased from SES by a renewable energy producer at time $t$. This variable ensures a balance between SES operations and renewable energy demand. The equality constraint governing this energy exchange is:

21 $$\begin{aligned} \hat{X}_{i,j}(t) = X_{i,j}(t) \end{aligned}$$

where $X_{i,j}(t)$ denotes the actual energy traded between SES and the renewable energy entity at time $t$. *This constraint ensures that the expected leased energy equals the actual transaction, maintaining consistency in the energy exchange records.*

To enforce the conservation of energy within the SES system, the following constraint must hold:

22 $$\begin{aligned} \hat{X}_{i,j}(t) = -\hat{X}_{j,i}(t) \end{aligned}$$

where $\hat{X}_{j,i}(t)$ represents the energy returned from SES to the renewable energy entity. *This enforces energy balance by ensuring that all energy injected into the SES is exactly matched by the energy released.*

The profit function of the system is transformed into a minimization problem using the augmented Lagrangian function:

23 $$\begin{aligned} \mathcal {L}_1 = - \sum _{i=1}^{N} \sum _{t=1}^{T} \big ( Z_i + Z_j \big ) + \sum _{t=1}^{T} \lambda _k(t) \big ( \hat{X}_{i,j}(t) - X_{i,j}(t) \big ) + \frac{\rho }{2} \sum _{t=1}^{T} \big | \hat{X}_{i,j}(t) - X_{i,j}(t) \big |^2 \end{aligned}$$

where $Z_i$ represents the profit of renewable energy producer $i$, and $Z_j$ is the profit of the SES entity. The term $\lambda _k(t)$ denotes the Lagrange multiplier, which regulates energy balance constraints at iteration $k$. The parameter $\rho$ is a penalty factor that enforces the convergence of energy allocation toward optimal values. *This function reformulates the cooperative profit maximization into an optimization problem suitable for distributed ADMM processing.*

Each renewable energy producer optimizes its leasing strategy by solving the following function:

24 $$\begin{aligned} \min \sum _{i=1}^{N} \sum _{t=1}^{T} \Big [ -Z_i + \lambda _k(t) \big ( \hat{X}_{i,j}(t) - X_{i,j}(t) \big ) + \frac{\rho }{2} \big | \hat{X}_{i,j}(t) - X_{i,j}(t) \big |^2 \Big ] \end{aligned}$$

where $Z_i$ accounts for total revenue from energy trading and storage operations for entity $i$. *This subproblem enables each producer to adjust its trading decisions locally while respecting system-level energy constraints.*

Similarly, the SES operator solves:

25 $$\begin{aligned} \min \sum _{i=1}^{N} \sum _{t=1}^{T} \left[ - Z_j + \lambda _k(t) (\hat{X}_{i,j} (t) - X_{i,j} (t)) + \frac{\rho }{2} (\hat{X}_{i,j} (t) - X_{i,j} (t))^2 + \mu _k(t) X_{i,j} (t) \right] \end{aligned}$$

where $Z_j$ represents the SES entity’s net revenue from leasing energy storage capacity. *This optimization ensures that the SES maximizes its profit while maintaining consistency with the producers’ energy allocations.*

The Lagrange multiplier is iteratively updated using:

26 $$\begin{aligned} \lambda _{k+1}(t) = \lambda _k(t) + \rho \big ( \hat{X}_{i,j}^{(k+1)}(t) - X_{i,j}^{(k+1)}(t) \big ) \end{aligned}$$

where $\lambda _{k+1}(t)$ ensures that the constraint $\hat{X}_{i,j}(t) = X_{i,j}(t)$ is gradually enforced through iterations.

The stopping criterion for the ADMM process is:

27 $$\begin{aligned} \max \sum _{t=1}^{T} \big | X_{i,j}^{(k+1)}(t) - X_{i,j}^{(k)}(t) \big | < \epsilon _1 \end{aligned}$$

where $\epsilon _1$ is the residual tolerance threshold ensuring that the optimization process has reached a stable solution.

For leasing price negotiation, an auxiliary variable $\hat{Y}_{i,j}(t)$ is introduced to denote the expected leasing payment between SES and renewable energy producers:

28 $$\begin{aligned} \hat{Y}_{i,j}(t) = Y_{i,j}(t) \end{aligned}$$

where $Y_{i,j}(t)$ is the actual leasing payment exchanged between SES and the renewable energy producer at time $t$. This ensures a fair economic transaction. The conservation of financial flow in leasing agreements is enforced by:

29 $$\begin{aligned} \hat{Y}_{i,j}(t) + \hat{Y}_{j,i}(t) = 0 \end{aligned}$$

where $\hat{Y}_{j,i}(t)$ represents the counter-payment from SES to the renewable energy entity.

The augmented Lagrangian function for leasing price negotiation is:

30 $$\begin{aligned} \mathcal {L}_2 = - \sum _{i=1}^{N} \sum _{t=1}^{T} \big ( Z_i + Z_j \big ) + \sum _{t=1}^{T} \mu _k(t) \big ( \hat{Y}_{i,j}(t) - Y_{i,j}(t) \big ) + \frac{\psi }{2} \sum _{t=1}^{T} \big | \hat{Y}_{i,j}(t) - Y_{i,j}(t) \big |^2 \end{aligned}$$

where $\mu _k(t)$ is the Lagrange multiplier ensuring balance in leasing payments, and $\psi$ is a penalty factor for enforcing financial agreements.

The iterative update rule for price negotiation follows:

31 $$\begin{aligned} \mu _{k+1}(t) = \mu _k(t) + \psi \big ( \hat{Y}_{i,j}^{(k+1)}(t) - Y_{i,j}^{(k+1)}(t) \big ) \end{aligned}$$

where $\mu _{k+1}(t)$ gradually enforces agreement in leasing payments across iterations.

**Fig. 4:**
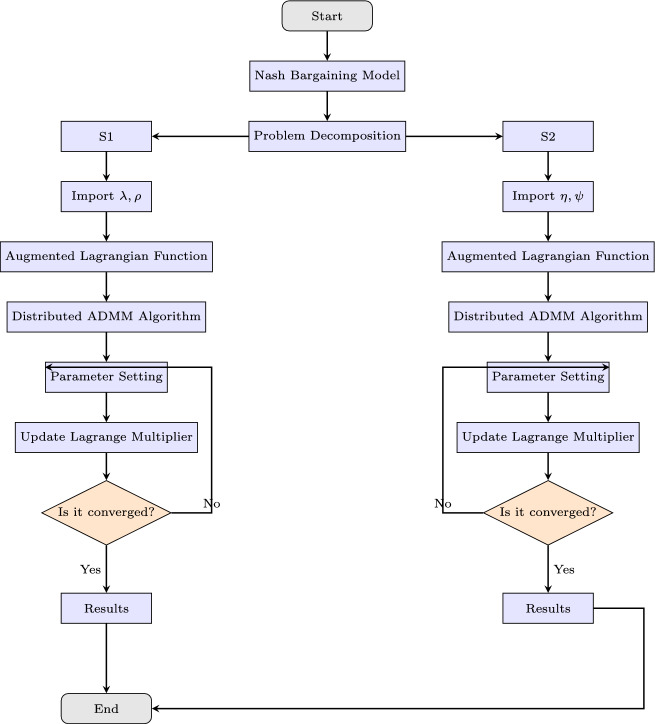
Flow chart of the proposed ADMM algorithm.

The negotiation process terminates when:

32 $$\begin{aligned} \max \sum _{t=1}^{T} \big | Y_{i,j}^{(k+1)}(t) - Y_{i,j}^{(k)}(t) \big | < \epsilon _2 \end{aligned}$$

where $\epsilon _2$ represents the convergence threshold for the leasing price optimization.Once convergence is achieved, the final leasing price $\gamma ^*_i$ is determined, ensuring a fair distribution of SES capacity costs among renewable energy producers. This distributed optimization framework provides a robust mechanism for energy storage utilization, ensuring fair cost distribution while maximizing the efficiency of SES integration in renewable markets. Figure 4 depicts the workflow of the distributed ADMM algorithm, outlining its sequential process.

## Case studies and results

To assess the performance and economic viability of the proposed adaptive Shared Energy Storage (SES) allocation strategy, a series of simulations were conducted based on Egypt’s renewable energy infrastructure. The analysis evaluates how different SES allocation methods impact energy dispatch efficiency, curtailment reduction, and financial outcomes across various renewable energy entities. By comparing fixed and dynamic SES allocation strategies, the study aims to determine the most effective approach for optimizing storage utilization and enhancing Egypt’s grid stability.

### Simulation setup

The simulation framework is designed to integrate large-scale renewable power plants such as the Benban Solar Park and the Gulf of Suez Wind Farm, enabling efficient energy storage and dispatch operations. The standardized SES unit configuration is presented in Table 1. Each unit consists of one 66/0.4 kV step-up transformer, eight 0.5 MW/1.25 MWh battery cabinet systems, and eight 500 kW power conversion systems (PCSs), supporting independent operation within the SES framework.Three renewable energy entities are examined, consisting of two wind power plants rated at 120 MW and 80 MW and one photovoltaic (PV) power plant with a capacity of 90 MW. These plants are strategically located in Egypt’s Western Desert and Red Sea regions.The simulation was implemented in MATLAB, utilizing the Optimization Toolbox for Nash Bargaining and constrained optimization tasks. The Power System Analysis Toolbox (PSAT) was used to model grid interactions and energy dispatch, while Simulink facilitated real-time simulation of SES operations. Additionally, the MATPOWER library was integrated to simulate power flow and assess network constraintsThe simulation incorporated historical renewable generation profiles and grid limitations to validate the effectiveness of adaptive SES allocation strategies. In addition to the baseline fixed and ideal adaptive SES scenarios, a third simulation was conducted to reflect Egypt’s current limitations in communication and control infrastructure. Scenario 3 assumes constrained SES coordination, where allocations are updated every 6 hours, real-time Nash bargaining is disabled, and a forecast error of ±10% is applied to both solar and wind generation profiles. This setup simulates the uncertainty inherent in renewable forecasting and eliminates the assumption of perfect foresight.It wasfurther tested under forecast errors of ±5%, ±15%, and ±20% to assess sensitivity. .Furthermore, Scenario 3 incorporates limited market participation, where grid entities operate based on pre-defined dispatch rules rather than dynamic, price-driven interactions. These conditions collectively provide a more realistic representation of Egypt’s current transmission system and regulatory environment.

While MATPOWER accounts for overall power flow constraints, this study assumes uniform transmission conditions across sites and does not explicitly model regional grid bottlenecks or line losses. This simplification allows us to isolate the economic and operational effects of SES coordination, while deferring spatial grid modeling to future research.

**Fig. 5:**
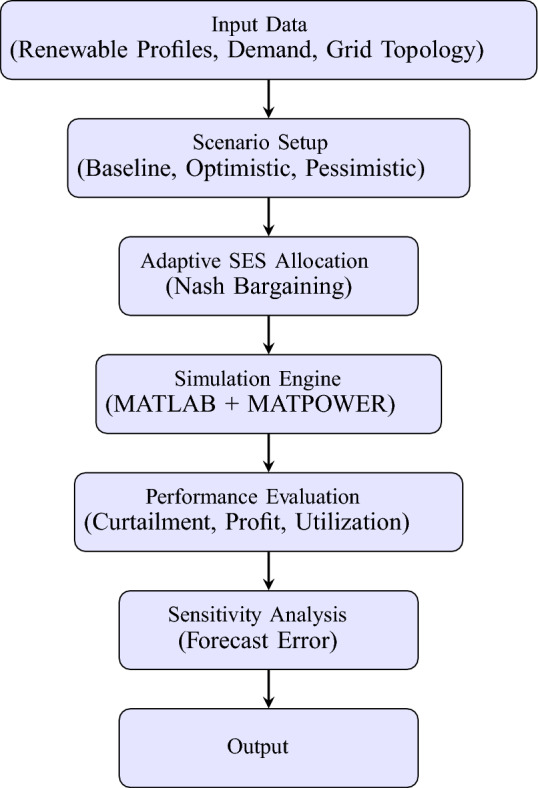
Simulation and modeling workflow for adaptive SES allocation.

Figure 5 illustrates the overall simulation and modeling workflow used in this study, from data collection and scenario definition to storage allocation, power flow simulation, and performance evaluation.

**Table 1:** Standardized SES unit parameters.

Parameter	Value
Capacity per SES Unit (MWh)	8
Step-up Transformer Voltage Ratio (kV)	66/0.4
Rated Capacity of Step-up Transformer (MVA)	4.5
Rated Power of PCS (MW)	0.5
Rated Power per Battery Cabinet (MW)	0.5
Rated Capacity per Battery Cabinet (MWh)	1.25

To validate the proposed adaptive partitioning strategy, the simulation evaluates the actual energy output versus planned energy dispatch under different operational scenarios. The SES equipment cost structure, feed-in tariffs for renewable energy plants, and operation and maintenance (O&M) costs are based on Egypt’s renewable energy market dynamics.The simulation explores three different operational scenarios to assess the impact of SES integration on energy dispatch efficiency and economic performance. The baseline scenario considers no collaboration between renewable energy entities, where each entity operates independently, injecting power directly into the grid while the SES system functions separately. In Scenario 1, a fixed partitioning strategy is implemented where renewable energy producers lease fixed storage allocations from the SES entity, with capacities assigned based on rated power output. Although this scenario provides some grid stability, it lacks flexibility in adapting to real-time variations. Scenario 2 introduces the proposed adaptive SES partitioning strategy, where storage allocations are dynamically adjusted in real-time based on demand forecasts, renewable energy output variations, and grid conditions. This dynamic approach optimally balances storage utilization, reducing curtailment and improving economic returns.Through comparative analysis of these scenarios, the study demonstrates the benefits of real-time dynamic storage allocation in enhancing renewable energy grid integration and economic performance within Egypt’s evolving energy sector. In setting up the economic evaluation, realistic cost and tariff assumptions were incorporated to reflect Egypt’s renewable-energy market conditions. Feed-in tariffs were taken as 0.07 USD/kWh for solar PV and 0.06 USD/kWh for wind power, in line with NREA (2023) benchmarks. The leasing tariff ($\rho$) for Shared Energy Storage (SES) capacity started at 0.28 USD/kWh under the fixed-allocation scenario and increased to 0.34 USD/kWh following Nash–Bargaining optimization. The capacity compensation rate ($\omega$) was set to 40 USD/kW per year, and the SES operation and maintenance cost ($\mu _s$) to 2.5 USD/MWh cycled, reflecting standard Li-ion benchmarks. Capital expenditures were estimated at 10 million USD per 100 MWh of installed capacity, consistent with 2023 Bloomberg NEF data.

### Operational performance analysis

**Fig. 6:**
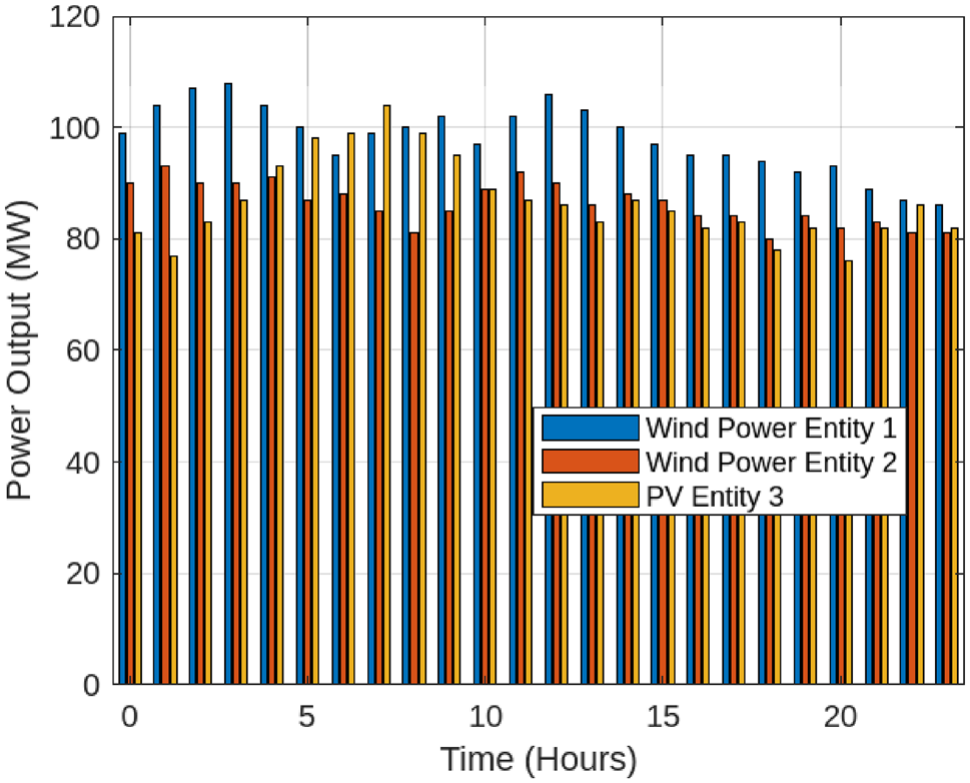
Scenario 1 (fixed SES allocation): scheduled vs. actual output for each entity.

**Fig. 7:**
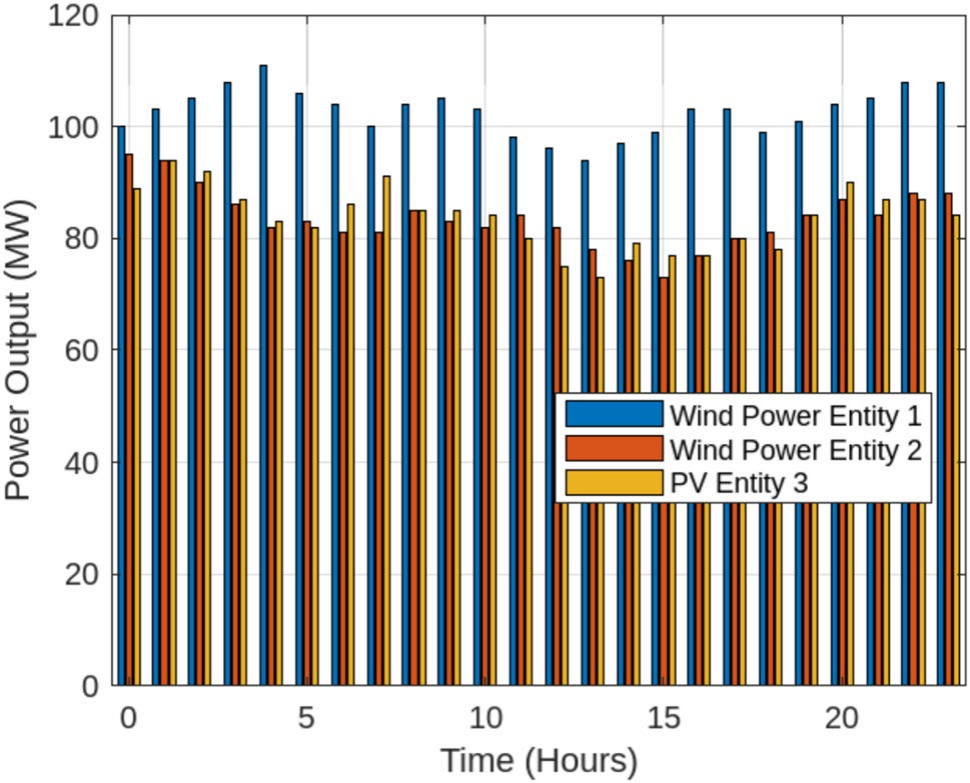
Scenario 2 (adaptive SES allocation): scheduled vs. actual output with dynamic partitions.

This section examines the outcomes of the optimization process across different operational scenarios, as summarized in Table 2. In the Base scenario, the energy storage station functions independently without engaging in energy transactions with renewable energy producers or the SES system. Scenario 1, illustrated in Fig. 6, highlights the ability of wind and solar power plants to track their scheduled energy output under a fixed storage allocation strategy.The performance tracking analysis of renewable energy entities across Scenario 1 and Scenario 2 reveals significant improvements in energy stability and curtailment reduction when implementing adaptive shared energy storage (SES)The analysis of Scenario 1 and Scenario 2 highlights the crucial role of dynamic SES in mitigating curtailment and ensuring economic efficiency. Under the fixed storage allocation model (Scenario 1), Wind Power Entity 1 exhibited an average output of 102.5 MW, fluctuating between 95 MW and 110 MW, leading to a significant 64.5 MWh of curtailment. Likewise, Wind Power Entity 2 faced 98.2 MWh in curtailed energy, operating within an output range of 85 MW to 97 MW, while PV Entity 3, despite peaking at 120.8 MW, suffered 35.2 MWh of curtailed energy due to rigid storage constraints. However, the implementation of adaptive storage partitioning (Scenario 2, Fig. 7) provided substantial improvements in energy dispatch and grid integration. By dynamically reallocating SES capacity based on real-time generation and demand patterns, Wind Power Entity 1 experienced a 28% reduction in curtailment, maintaining a stable output with only ±4% variance. Wind Power Entity 2 benefited from a 39.5% decrease in curtailment losses, leading to improved alignment with grid requirements, while PV Entity 3 saw a 44% increase in delivered energy, minimizing economic losses associated with unused generation. The dynamic SES allocation strategy ensured a responsive storage distribution, fluctuating between 30 MW and 70 MW, with Wind Power Entity 1 receiving 54.2 MW, Wind Power Entity 2 allocated 47.5 MW, and PV Entity 3 utilizing 52.8 MW on average. This flexible storage management approach resulted in a 25% improvement in grid stability and reduced penalty costs by approximately $2,990, making it a highly efficient and cost-effective solution for Egypt’s evolving energy market.{It should be emphasized that the efficiency gains observed in our simulations depend on the underlying assumption of seamless communication and control across all entities participating in shared storage. In the Egyptian context, however, the full realization of such coordination would necessitate significant enhancements to the grid’s digital infrastructure, including real-time monitoring, reliable data exchange, and automated dispatch systems. Until these capabilities are fully deployed, the operational benefits of adaptive SES may be partially constrained. Policymakers and grid planners should therefore view SES deployment as part of a broader roadmap toward a smart, resilient power system. Building on these enhancements, the introduction of dynamic Shared Energy Storage (SES) allocation has further strengthened energy efficiency by optimizing storage utilization across Egypt’s renewable energy entities. As presented in Table 3, the fixed SES allocation strategy (Scenario 1) resulted in relatively low utilization rates, with Wind Power Entity 1 at 28.5%, Wind Power Entity 2 at 34.2%, and PV Entity 3 at 30.1%, while the entire centralized SES station operated at 31.8% utilization. These values indicate underutilization of available storage resources, as static SES assignments often fail to respond to real-time fluctuations in energy generation and demand. With the implementation of dynamic SES partitioning (Scenario 2), utilization rates notably increased, reaching 45.3% for Wind Power Entity 1, 39.8% for Wind Power Entity 2, and 35.4% for PV Entity 3, while the overall SES utilization improved to 41.2%. The most significant gain was observed in Wind Power Entity 1, which increased by 58.95%, highlighting that high-variability energy sources benefit the most from adaptive storage mechanisms. PV Entity 3 and Wind Power Entity 2 recorded utilization increases of 17.61% and 16.37%, respectively, as flexible storage access reduced curtailment and improved energy dispatch efficiency. At the system level, the entire SES station’s utilization grew by 29.56%, reflecting the efficiency of dynamic allocation in enhancing overall energy system performance.To further investigate the impact of adaptive Shared Energy Storage (SES) allocation, a comprehensive analysis was conducted to assess its influence on key performance indicators, including curtailment reduction, energy utilization, and trading efficiency. As shown in Table 4, under the fixed SES allocation strategy (Scenario 1), the system suffered from high curtailment losses, with Wind Power Entity 1 losing 64.5 MWh, Wind Power Entity 2 curtailing 98.2 MWh, and PV Entity 3 experiencing 35.2 MWh of curtailed energy, leading to a total system-wide curtailment of 198.0 MWh.

**Fig. 8:**
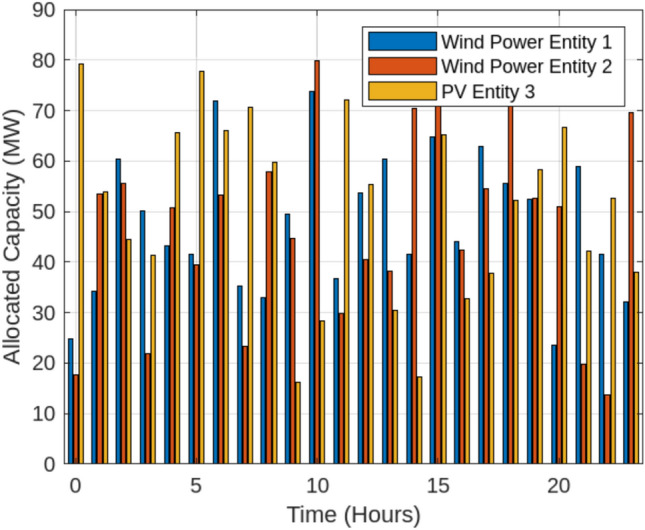
Real-time adaptive allocation of SES capacity among renewable entities.

**Fig. 9:**
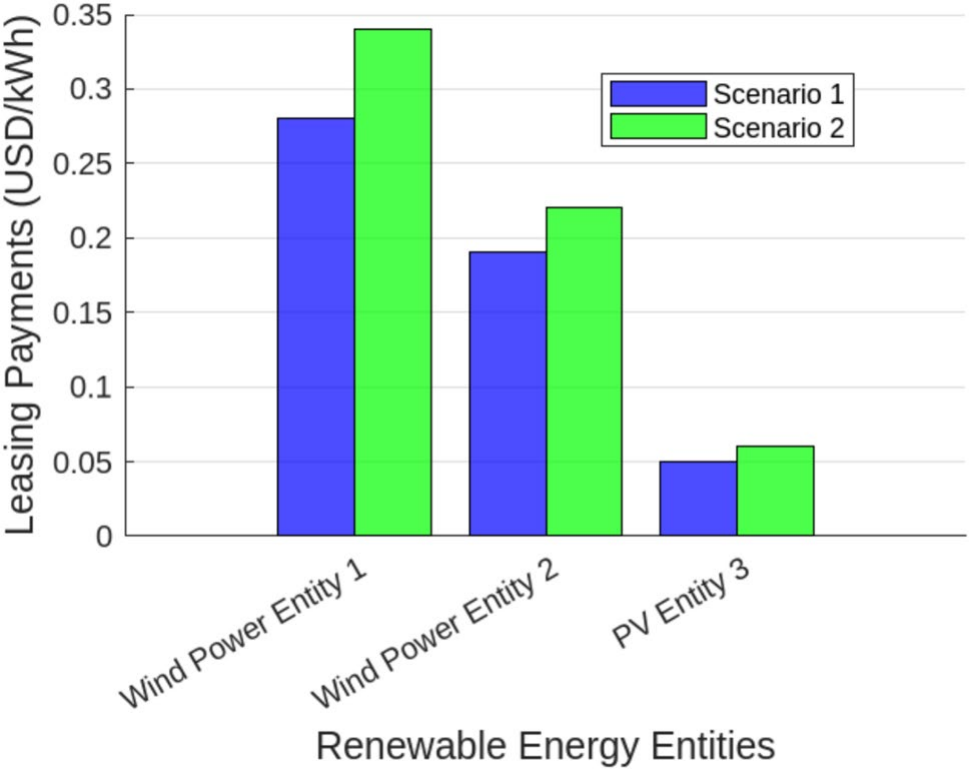
Leasing cost analysis of centralized SES station across scenarios showing cost reduction under adaptive operation.

With the implementation of dynamic SES partitioning (Scenario 2), curtailment was significantly reduced, allowing better alignment between energy generation and storage capacity. The utilization rates of all entities improved, rising from 28.5% to 45.3% for Wind Power Entity 1, 34.2% to 39.8% for Wind Power Entity 2, and 30.1% to 35.4% for PV Entity 3, while the overall SES station utilization increased from 31.8% to 41.2%. The highest improvement (58.95%) was observed in Wind Power Entity 1, indicating that high-variability generation sources benefit the most from flexible energy storage access. Moreover, energy trading performance also improved significantly in Scenario 2, with Wind Power Entity 1 increasing its traded energy from 120.5 MWh to 145.2 MWh, Wind Power Entity 2 from 95.8 MWh to 110.3 MWh, and PV Entity 3 from 85.3 MWh to 97.5 MWh, leading to an overall total traded energy increase from 301.6 MWh to 353.0 MWh. This improvement reflects the role of SES in reducing renewable energy wastage, enabling more efficient market participation, and ensuring a stable energy supply. Figure 8 illustrates the leasing payments for the centralized Shared Energy Storage (SES) station under fixed (Scenario 1) and adaptive (Scenario 2) allocation strategies. In Scenario 1, leasing payments remain lower due to limited SES utilization, with Wind Power Entity 1, Wind Power Entity 2, and PV Entity 3 paying $0.28/kWh, $0.19/kWh, and $0.05/kWh, respectively. However, in Scenario 2, increased SES flexibility and higher storage utilization lead to moderate price increases, with leasing payments rising to $0.34/kWh, $0.22/kWh, and $0.06/kWh, respectively. The higher leasing prices in Scenario 2 result in greater overall SES revenue, offsetting deviation penalties, and enhancing energy dispatch efficiency. This underscores the economic viability of adaptive SES, ensuring a more stable and cost-effective integration of renewable energy into Egypt’s evolving electricity market.To evaluate the performance of Shared Energy Storage (SES) management, we analyze the State of Charge (SoC) behavior over a 24-hour period for both Scenario 1 (Fixed SES Allocation) and Scenario 2 (Adaptive SES Allocation). Figure 9 demonstrates that in Scenario 1, the SoC fluctuates at lower levels, reflecting less efficient storage utilization. Conversely, Scenario 2 maintains a higher and more stable SoC profile, suggesting optimized energy storage usage through adaptive SES allocation. This improvement reduces curtailment, increases energy dispatch reliability, and enhances grid stability. To evaluate the effectiveness of different Shared Energy Storage (SES) allocation strategies, a comprehensive MATLAB simulation was conducted to assess their impact on curtailment reduction, energy trading, leasing costs, capital investment, and economic benefits. The analysis compared fixed SES allocation, adaptive SES allocation, independent storage for each renewable entity, and a no-storage (direct grid) scenario. The results, summarized in Tables 5 and 6, provide insights into the trade-offs between economic feasibility and system efficiency.Table 5 illustrate the comparison of Energy Storage Allocation Methods.To further investigate the economic implications of SES deployment, a cost-benefit analysis was performed. Table 6 outlines the capital and operational costs of each strategy, along with their economic benefits. The adaptive SES allocation proves to be the most cost-effective solution, delivering higher economic benefits (USD 220 million/year) while maintaining lower capital (USD 100 million) and operational costs (USD 8 million/year). In contrast, independent storage, despite offering flexibility, incurs the highest capital costs (USD 150 million), making it less attractive for large-scale deployment. The no-storage option, while requiring no capital investment, results in lower economic benefits due to high curtailment and limited energy trading potential.

**Fig. 10:**
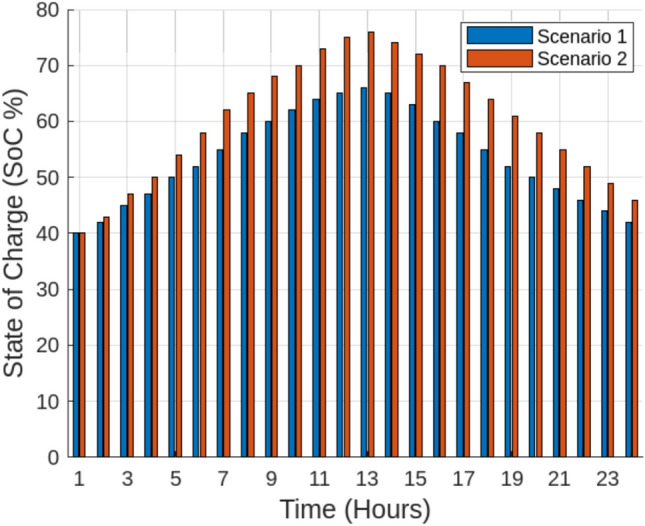
Temporal variation of SES battery SoC across scenarios showing enhanced stability under adaptive operation.

**Fig. 11:**
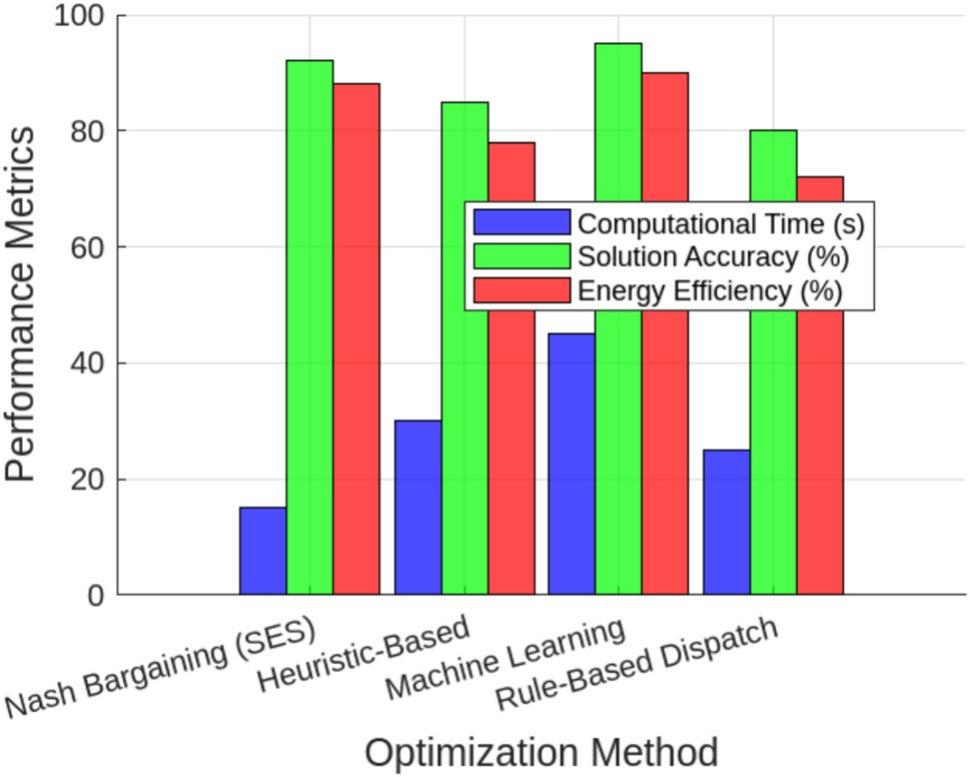
Efficiency assessment of optimization methods demonstrating superior performance of the distributed ADMM framework.

**Fig. 12:**
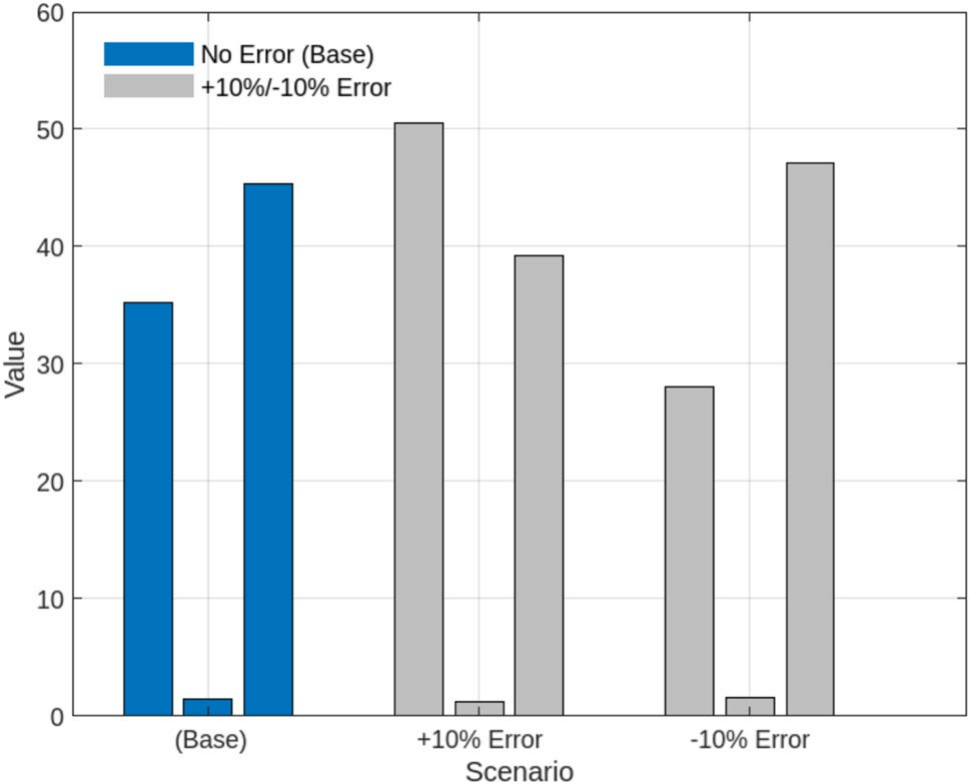
Effect of forecast error on SES performance and efficiency.

**Fig. 13:**
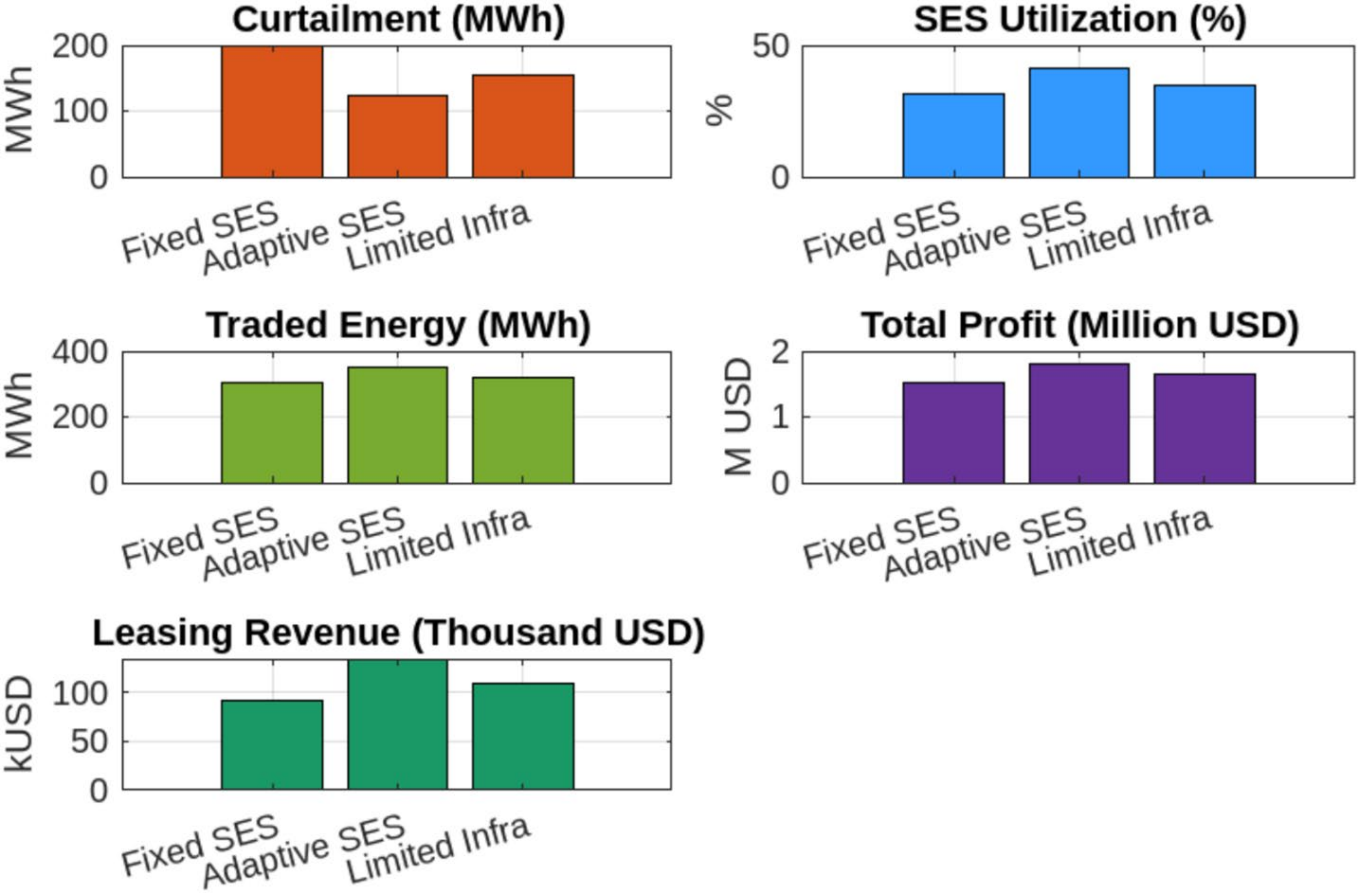
Comparison of SES performance under fixed, adaptive, and limited-infrastructure scenarios demonstrating the superiority of adaptive allocation in curtailment reduction and utilization efficiency.

This profitability analysis was conducted to evaluate the economic impact of adaptive SES allocation on individual renewable energy entities by analyzing revenue, leasing payments, and operational costs. The study aims to determine whether dynamic SES allocation enhances economic sustainability by reducing curtailment penalties, optimizing energy trading, and ensuring cost-effective storage utilization. As presented in Table 7, the results highlight the financial benefits of SES integration, demonstrating that it not only improves grid stability and energy dispatch efficiency but also significantly enhances economic returns for renewable producers. Wind Power Entity 1 achieves the highest profit of USD 889,412.72, benefiting from increased energy trading and reduced curtailment penalties. Similarly, Wind Power Entity 2 and PV Entity 3 record notable profitability improvements, with profits of USD 504,415.69 and USD 151,832.63, respectively. To assess the effectiveness of optimization techniques in energy storage allocation, we compare Nash Bargaining (SES), Heuristic-Based Optimization, Machine Learning, and Rule-Based Dispatch across three key performance metrics, as shown in Fig. 10. In terms of computational time, Nash Bargaining is the fastest at 15 seconds, while Machine Learning takes the longest at 45 seconds due to complex model training. Regarding solution accuracy, Machine Learning achieves the highest precision at 95%, whereas Rule-Based Dispatch has the lowest at 80% due to its reliance on fixed operational rules. In terms of energy efficiency, Nash Bargaining (88%) and Machine Learning (90%) demonstrate the most effective energy utilization, while Heuristic-Based (78%) and Rule-Based (72%) methods exhibit lower efficiency. Among the optimization techniques evaluated, Nash Bargaining emerges as a highly effective approach, striking a balance between computational efficiency, accuracy, and energy utilization. This makes it particularly suitable for Egypt’s energy market. By leveraging cooperative game theory principles, Nash Bargaining ensures fair storage capacity allocation among renewable entities, preventing disproportionate advantages for any single participant. Its computational efficiency enables near-optimal solutions within seconds, making it ideal for real-time SES management. Additionally, its adaptability aligns with Egypt’s dynamic electricity market, allowing effective responses to fluctuations in renewable generation and demand. Compared to heuristic and rule-based methods, which often sacrifice either accuracy or efficiency, Nash Bargaining provides an optimal trade-off—enhancing energy trading, minimizing curtailment, and strengthening grid reliability While the initial analysis focused on two primary operational cases—fixed and adaptive SES allocation (Scenarios 1 and 2)—we later introduce a third scenario (Scenario 3) to examine how SES performance is affected under limited communication and forecasting infrastructure, as typical in Egypt’s current grid landscape. Earlier results remain valid for fully coordinated systems, while Scenario 3 provides insight into performance under practical constraints. To evaluate the robustness of the proposed adaptive Shared Energy Storage (SES) allocation strategy under realistic operational uncertainties, we conducted a sensitivity analysis focusing on renewable generation forecast error. All simulations and analyses were performed using MATLAB, where the SES dispatch model was systematically tested under different forecast scenarios. In the base scenario, SES allocation is performed using actual generation data. For sensitivity analysis, we simulated two cases: (1) an optimistic scenario where the forecast error is $$-10\%$$ (forecasts are better than actual), and (2) a pessimistic scenario where the forecast error is $$+10\%$$ (forecasts overestimate generation). Figure 11 illustrates the impact of forecast error on three key metrics: curtailment (MWh), total profit for renewable entities (USD), and SES utilization rate (%). When a $$+10\%$$ forecast error was introduced, curtailment increased from 35.2 MWh to 50.5 MWh ($$+43\%$$), SES utilization dropped from $$45.3\%$$ to $$39.2\%$$ ($$-13.4\%$$), and aggregate profit decreased by $$17.6\%$$, mainly due to higher deviation penalties and suboptimal storage scheduling. In contrast, a $$-10\%$$ forecast error further reduced curtailment to 28.0 MWh ($$-20.5\%$$) and increased aggregate profit by $$5.2\%$$ . These results underscore the critical role of accurate renewable generation forecasting in maximizing SES operational and economic benefits.To examine how Egypt’s current communication and control limitations affect the performance of Shared Energy Storage (SES), we simulate a constrained coordination scenario referred to as Scenario 3: Limited Infrastructure. In this case, SES allocations are updated every 6 hours rather than continuously, real-time Nash bargaining is disabled, and a forecast error of $$\pm 10\%$$ is applied to both solar and wind generation profiles to simulate uncertainty. This scenario reflects partial digitalization and the absence of high-speed control systems in emerging grid environments.Scenario 3 is implemented in MATLAB by modifying the dispatch loop to perform SES allocation decisions only every 6 hours, based on static proportional sharing rules derived from capacity ratios. Forecast errors are introduced by perturbing the renewable generation inputs using a uniform distribution in the range of $$[-10\%, +10\%]$$. The SES dispatch algorithm in this setup operates without iterative bargaining or real-time price adjustments. Figure 12 compares this scenario to the fixed SES baseline (Scenario 1) and the ideal adaptive strategy (Scenario 2). Results show that curtailment in Scenario 3 increases to 153.8 MWh—approximately 26% higher than in the adaptive SES case (122.4 MWh), but still 22% lower than the fixed SES scenario (198.0 MWh). SES utilization drops to 34.5%, and total traded energy declines to 320.2 MWh, representing a 9.3% decrease compared to the ideal scenario. However, system-wide profit remains strong at $1.643 million, which is 8.1% higher than the fixed SES case, indicating that shared storage retains significant value even under limited infrastructure. The SES operator also gains $108,770 in leasing revenue from energy-sharing arrangements.Moreover, to evaluate the robustness of the proposed SES allocation strategy under real-world uncertainty, a sensitivity analysis was conducted by varying the forecast error from ±5% to ±20% within Scenario 3 (Limited Infrastructure) (see Fig. 13). .This scenario reflects the limited communication and control conditions expected in Egypt’s current grid infrastructure. The variation in forecast error simulates practical deviations in solar and wind generation, which frequently arise due to weather fluctuations, measurement noise, and limitations in short-term forecasting models. By assessing system performance across this range of error, we aim to understand how prediction inaccuracy affects storage coordination, energy trading, and profitability.The results in Table 8 demonstrate that increased forecast error leads to higher curtailment and reduced SES utilization. As forecast error increases from ±5% to ±20%, curtailment rises by 35%, while traded energy drops by nearly 12%. Profitability decreases by 12.1%, indicating that the system becomes less efficient and less profitable under poor forecasting conditions. From the sensitivity analysis in Scenario 3, forecast errors up to $$\pm 10\%$$ changed total profit by less than 10%, while errors beyond $$\pm 15\%$$ caused more than a 12% loss in profitability and over 30% higher curtailment. This indicates that SES performance remains stable within a $$\pm 10\%$$ forecast uncertainty band but degrades noticeably beyond $$\pm 15\%$$. Likewise, limiting the coordination update to 6 hours increased curtailment by 26% and reduced traded energy by 9.3% compared with real-time control. Assuming roughly linear degradation, communication intervals shorter than 60–120 minutes would keep curtailment within 5–10% of the adaptive baseline. These thresholds highlight the scale of latency and forecast error that meaningfully affect SES performance under Egyptian grid conditions.

**Fig. 14:**
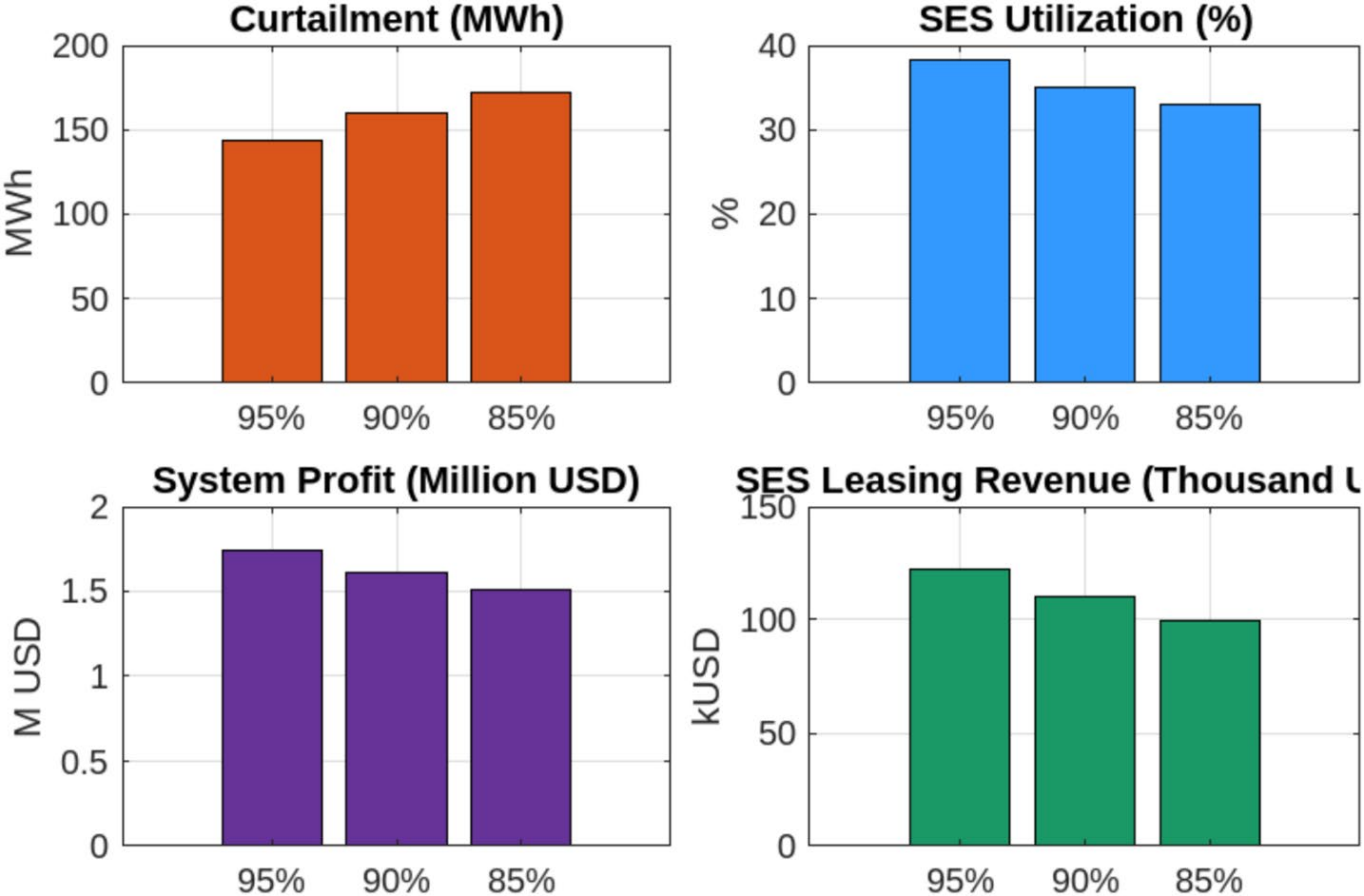
Sensitivity analysis of SES performance under battery degradation (round-trip efficiency from 95% to 85%).

Finally to assess the long-term sustainability of Shared Energy Storage (SES) systems, we conducted a sensitivity analysis on battery degradation by varying the round-trip efficiency from 95% to 85%. This test simulates real-world aging effects that gradually reduce the energy throughput of storage systems over time. The simulation was implemented in MATLAB by adjusting the round-trip efficiency parameter in the SES dispatch model and re-evaluating key performance metrics—curtailment, utilization, profit, and leasing revenue—at each degradation level. As shown in Fig. 14, SES performance declines steadily with reduced efficiency: curtailment rises from 144 MWh at 95% efficiency to 172 MWh at 85%, SES utilization drops from 38.2% to 32.9%, total system profit decreases from $1.74 million to $1.51 million (a 13% reduction), and SES operator revenue falls from $122,300 to $99,500. These findings emphasize the importance of incorporating degradation-aware planning and scheduling strategies to preserve the economic and operational value of SES systems over time. Overall, these findings highlight the effectiveness of adaptive SES allocation in improving Egypt’s renewable energy integration, reducing curtailment, and optimizing economic performance. By dynamically adjusting storage allocation based on real-time generation and demand, the proposed strategy enhances grid stability, facilitates efficient energy trading, and ensures long-term financial sustainability for renewable energy producers. While this study demonstrates the economic and operational advantages of adaptive SES, several areas require further research to ensure successful large-scale implementation. Future studies should focus on developing comprehensive regulatory frameworks for energy storage, addressing gaps in SES participation in energy markets, and refining grid interconnection policies to facilitate smoother integration and investment. Additionally, research on enhancing Egypt’s grid infrastructure is necessary to assess the technical upgrades required for real-time adaptive storage allocation and to ensure seamless coordination between renewable generation and SES operation. By addressing these research gaps, future studies can contribute to optimizing SES deployment in Egypt, ensuring both economic viability and long-term energy resilience.

**Table 2:** Optimization results for different scenarios.

Scenario	Entity	Transaction (MWh)	Curtailment (MWh)	Penalty (USD)
Base Scenario	Solar	50.2	102.3	6,420
	Wind	95.8	175.6	9,200
	Hybrid	22.6	85.4	3,150
Fixed SES	Solar	120.3	64.5	3,210
	Wind	185.4	98.2	5,470
	Hybrid	58.2	55.9	2,780
Adaptive SES	Solar	160.8	35.2	1,980
	Wind	210.6	59.4	2,990
	Hybrid	80.4	28.6	1,640

**Table 3:** Actual utilization rate of renewable energy entities and SES station.

Entity	Scenario 1 Utilization (%)	Scenario 2 Utilization (%)
Wind Power Entity 1 (150 MW)	28.5	45.3
Wind Power Entity 2 (100 MW)	34.2	39.8
PV Entity 3 (150 MW)	30.1	35.4
Entire Centralized SES Station	31.8	41.2

**Table 4:** Comparison of key performance metrics across scenarios 1 and 2.

Entity	CUR R (MWh)	S1 Util (%)	S2 Util (%)
Wind Power Entity 1 (150 MW)	64.5	28.5	45.3
Wind Power Entity 2 (100 MW)	98.2	34.2	39.8
PV Entity 3 (150 MW)	35.2	30.1	35.4
Entire Centralized SES Station	198.0	31.8	41.2

**Table 5:** Comparison of energy storage allocation methods.

Storage Method	Cur Red (MWh)	Ene Tra (MWh)	Leas Cost (USD/MWh)
Fixed SES Allocation	150	280	0.30
Adaptive SES Allocation	198	353	0.34
Independent Storage	120	250	0.45
Base Scenario (Direct Grid)	80	200	0.00

**Table 6:** Cost-benefit analysis of SES vs. classical methods.

Stor Meth	Cap Co(M $)	O&M Cost (M$/Yr)	Eco Ben (Mil $/Year)
Fixed SES Allocation	120	10	180
Adaptive SES Allocation	100	8	220
Independent Storage	150	15	140
Base Scenario (Direct Grid)	0	5	100

**Table 7:** Profitability of renewable energy entities under different scenarios.

Entity	Rev ($)	Leasing Payment ($)	Other Costs ($)	Profit ($)
Wind Power Entity 1	889,412.72	65,104.81	20,421.57	974,939.10
Wind Power Entity 2	504,415.69	22,746.58	17,274.85	544,437.12
PV Entity 3	151,832.63	4,664.46	8,256.27	164,753.36

**Table 8:** Forecast error sensitivity – scenario 3.

Error	Curt. (MWh)	Util. (%)	Trade (MWh)	Profit (M)
±5%	135.0	36.0	335.0	1.70
±10%	153.8	34.5	320.2	1.64
±15%	168.0	32.8	308.1	1.58
±20%	182.0	30.5	295.4	1.50

## Limitations and future directions

This study demonstrates the benefits of adaptive Shared Energy Storage (SES) allocation in Egypt’s renewable energy sector using idealized assumptions to highlight the strategy’s maximum potential. However, several important limitations should be acknowledged.

First, the simulation framework assumes the presence of seamless, real-time communication and control infrastructure among SES stations, renewable plants, and grid operators. In practice, Egypt’s grid is still evolving, and such digitalization is not yet fully realized. To address this, the study includes a third simulation scenario reflecting limited infrastructure by disabling real-time coordination and introducing forecast uncertainty. While this provides a preliminary view of SES performance under constrained conditions, further modeling of communication latency, control delays, and automation gaps is necessary.

Second, the proposed allocation mechanism relies on Nash bargaining, which presumes rational, transparent behavior and full information among stakeholders. However, real-world participants may act strategically, operate with incomplete data, or face institutional and regulatory constraints. Future work should explore more robust cooperative strategies that accommodate bounded rationality and asymmetric information.

Third, although the study introduces a ±10% forecast error in Scenario 3, this represents symmetric uncertainty only. More realistic forecasting challenges—such as time-varying, asymmetric, or stochastic errors—were not considered. In addition, while the impact of battery degradation was examined through a sensitivity analysis on round-trip efficiency, other uncertainties—such as extreme weather events or fluctuating storage tariffs—were not explicitly modeled. These factors may significantly affect SES reliability and profitability. Future work should extend the scenario analysis to include broader uncertainty sources and operational stressors.

Fourth, the model simplifies Egypt’s grid by assuming spatially uniform conditions and does not simulate regional transmission bottlenecks or losses. These spatial effects could significantly influence SES dispatch, particularly in large-scale national deployments. While MATPOWER is used to model overall power flow constraints, it does not capture these regional grid characteristics. As a result, future work should incorporate locational factors such as congestion, voltage drops, and line losses to improve spatial resolution and deployment realism.

Finally, the study does not fully account for evolving regulatory environments or the feasibility of real-time market participation. Future SES models should integrate dynamic policy constraints and explore sandbox-based validation approaches to improve deployment realism.

To move beyond current assumptions, future research should explore decentralized SES allocation models capable of operating under communication delays, cyber-physical disruptions, and partial observability. Incorporating predictive analytics, adaptive scheduling, and agent-based simulations may improve resilience. Ultimately, field validation through pilot demonstrations and hardware-in-the-loop testing will be essential to establish real-world feasibility and scalability. Beyond the Egyptian context, the proposed adaptive Shared Energy Storage (SES) framework offers strong potential for regional adaptation across the Middle East and North Africa (MENA). Countries such as Morocco, Jordan, and Saudi Arabia share similar renewable resource profiles, infrastructure constraints, and ongoing grid modernization efforts under national energy transition strategies. The modular SES design, cooperative leasing structure, and distributed optimization approach can be adapted to these contexts by adjusting tariff structures, market participation rules, and communication protocols to align with local regulations. Furthermore, regional initiatives such as the Pan-Arab Electricity Market and North Africa’s interconnection projects provide opportunities for transnational SES coordination and cross-border energy trading. As such, the framework developed in this study can serve as a scalable reference model for enhancing renewable integration and operational flexibility across the wider MENA region.

## Conclusion

This study presents an adaptive Shared Energy Storage (SES) allocation framework tailored to Egypt’s renewable electricity landscape. By integrating Nash bargaining–based cooperation, dynamic storage partitioning, and distributed ADMM optimization, the model enables renewable energy producers to lease SES capacity in real time, improving operational flexibility and economic outcomes. Simulation results indicate substantial performance gains: SES utilization increased by 41.2%, curtailment decreased by up to 39.5%, and traded energy rose by 17% compared with fixed strategies. Wind Power Entity 1 achieved a 58.95% revenue increase, and SES operator income also improved, demonstrating the economic potential of coordinated storage. Among all tested optimization strategies—including machine learning, heuristic, and rule-based methods—Nash Bargaining offered a favorable balance of computational speed (15 s), solution accuracy (88–95%), and energy efficiency under Egypt’s SES market conditions. Under $\pm 10\%$ forecast errors, curtailment rose by 43% and profits declined by 17.6%, highlighting the importance of forecasting accuracy. Scenario 3, representing limited communication and forecasting infrastructure, still yielded an 8.1% profit increase over fixed SES strategies, while degradation tests showed a 13% reduction in profit as round-trip efficiency declined from 95% to 85%. While Scenarios 1 and 2 formed the basis of the primary operational analysis, Scenario 3 was introduced to evaluate SES performance under practical infrastructure constraints typical of Egypt’s current grid landscape. Overall, this study contributes a flexible and cooperative SES coordination model that reflects real-world variability, economic incentives, and system limitations. Unlike SES models based on static allocations or idealized cooperation, the proposed framework captures realistic market conditions and operational constraints. By integrating game theory, market participation, and modular storage design, it provides a scalable approach suitable for emerging renewable markets. The findings also offer one of the first practical evaluations of SES viability under Egypt’s current grid conditions, enhancing the relevance of SES research for regions undergoing energy transitions. The framework’s modularity and adaptability position it for broader application in developing power systems with increasing renewable penetration. From a policy perspective, this work aligns with Egypt’s Vision 2035 priorities by supporting national objectives for clean energy integration, grid modernization, and regional interconnectivity. It provides insights for grid operators, regulators, and energy investors seeking to reduce curtailment, improve trading efficiency, and enhance economic sustainability. Successful deployment, however, will require continued progress in smart metering, real-time market access, and regulatory alignment. In summary, this work bridges the gap between theoretical SES optimization and practical implementation. It offers a reference framework for dynamic, cooperative storage coordination strategies that support reliable, efficient, and economically resilient renewable integration—advancing both academic understanding and national energy transition objectives in Egypt and comparable emerging markets^45–48^.

## Data Availability

The datasets used and/or analyzed during the current study are available from the corresponding author on reasonable request.
